# Are some neutral liners more neutral than others? An ex vivo morphological analysis of acetabular liners classified as “neutral”

**DOI:** 10.2340/17453674.2024.41946

**Published:** 2024-10-11

**Authors:** José Á OCHOA, Perttu S NEUVONEN, Jari HYTTINEN, Jari VIIK, Antti P ESKELINEN

**Affiliations:** 1 Faculty of Medicine and Health Technology, Tampere University, Tampere; 2 Coxa Hospital for Joint Replacement, Tampere, Finland

## Abstract

**Background and purpose:**

In contemporary total hip replacement (THR), dislocation is one of the most common complications. At our institution, the cause of an increase in the dislocation rate was recently reported to be reduced head coverage of a newly introduced neutral liner. We therefore aimed to ascertain whether differences exist in articulating head coverage between the various neutral liners used in contemporary THR. A secondary aim was to utilize coverage measurements to develop a new liner coverage classification.

**Methods:**

The articulating head coverage of 25 modular neutral polyethylene liners used in 6 uncemented cup designs from 4 major manufacturers was evaluated. The measurements were performed in a metrology laboratory and a mathematical model was developed to calculate coverage of the articulating surfaces. Further, 1 “elevated rim” liner and 1 “face changing liner” were included to develop a new liner coverage classification.

**Results:**

The articulating head coverage among the studied liners ranged from 167.7° to 194.8°, corresponding to a variation of 27.1°. The variations with different cup and head sizes within each design were smaller (from 1.0° to 5.6°) than those between different designs. Each of the liner designs offered distinct coverage, even though they were all classified as neutral. Based on measurements, a set of descriptive parameters to discriminate different liners in terms of coverage was created.

**Conclusion:**

We showed that all neutral liners are not equal – instead, they clearly varied in terms of their actual coverage design. We suggest our set of descriptive parameters called “hemispheric coverage index values” be used in discriminating the differences in liner coverage.

Dislocation is one of the most common complications after primary total hip replacement (THR) with an incidence varying between 2% and 5.1% [1-6]. Dislocation can cause substantial patient morbidity, readmissions, and reoperations, which subsequently increase the cost of care [1,5,7].

Many patient-, surgical technique-, and implant-related risk factors for dislocation have been identified [8-10]. Of these, the articulating components of a hip prosthesis—the femoral head and the acetabular liner—have a direct impact on hip stability. Both the size of the femoral head and the coverage of the acetabular liner affect jumping distance (JD), which is a measurement that represents the lateral translation of the femoral head center on the liner to generate a dislocation. The bigger the JD, theoretically, the more stable the joint, and the more difficult it is for the articulating components to dislocate [11].

There are several types of acetabular liners available from different manufacturers with considerable variations in geometry. However, manufacturers do not publicly share the real coverage of the implants. In a recent clinical study from our institution, the coverage of polyethylene (PE) liners classified as “neutral” differed from one manufacturer to another. The study also revealed that smaller coverage led to an increasing incidence of dislocations [6]. At present, the technical specifications provided by the manufacturers are not adequate to describe liners’ biomechanical behavior and the literature on liner coverage is scarce [10-14].

Our primary aim was to assess whether there are coverage differences in the neutral liner designs from 4 major hip implant manufacturers used in contemporary THR. Our secondary aim was to utilize the measurements acquired to establish a new liner coverage classification.

## Methods

### Design

We evaluated 25 neutral PE liners from 4 different manufacturers. For the evaluation, X3 liners for the Trident and Trident II cup systems (Stryker, Kalamazoo, MI, USA), R3 XLPE liners for the R3 cup system (Smith & Nephew, Watford, UK), Marathon liners for the Pinnacle cup system (DePuy Synthes, Raynham, MA, USA), E1 liners for the G7 cup system (Zimmer Biomet, Warsaw, IN, USA), and Longevity liners for the Continuum cup system (Zimmer Biomet, Warsaw, IN, USA) were selected to represent the liners of the cementless, porous-coated modular cup systems, which currently dominate the market in Finland and internationally [1,5,6].

There are alternative liners available for the Pinnacle cup (ALTRX), as well as for the G7 and Continuum cups (ArCom, ArComXL and Vivacit-E). We included 1 Pinnacle ALTRX and 1 G7 ArComXL liner to check whether the coverage of the alternate liners was in line with Marathon and E1 liners.

The liners for 3 different cup sizes (46 mm, 52 mm, 58 mm) were measured. The cup sizes were selected in 6 mm steps around 52 mm, which is the most common cup size used at our institution. Liners for 32 mm and 36 mm head sizes were evaluated in cup sizes of 52 mm and 58 mm. As there is variation between brands of head sizes available in size 46 mm, the largest head size available was chosen (28 mm for the Marathon, R3, and Continuum Longevity, and 32 mm for the X3 and G7 E1). The Marathon liner in size 52/36 mm was replaced by the ALTRX, as there is no Marathon liner available in that size. The ArComXL liner was measured in size 58/32 mm.

### Measurement of geometry

The geometry of the articulating surfaces of the liners was measured using a coordinate measuring machine (CMM, Metrology Laboratory Services, Tampere University, Tampere, Finland). CMM is an essential tool in metrology laboratories for accurately describing physical objects by measuring discrete points on the surface of the object with a probe. A SIP CMM5 (Société d’Instruments de Précision, Geneva, Switzerland) was used (measurement uncertainty: MPEE = 0.8 µm + 1.2 µm/m, MPEP = 0.8 µm), with a Renishaw SP25 (Renishaw, Wotton-under-Edge, UK) touch probe (ø 3 mm, ruby ball), a Renishaw PH10 rotating measuring head, and Metrolog X4 software (Metrologic Group, Isère, France). Finally, the SolidWorks 2019 (SolidWorks, Waltham, MA, USA) drawing tool was used for 3D figure representation and the technical drawings. CMM was calibrated according to the guidelines provided in ISO 10360. Further, measurements taken by the CMM were validated using a Control Reference (Calibration Probe for SIP CMM5). Each measurement was performed twice and a mean surface was generated. If any of the measurement points deviated more than 4 µm compared with the generated geometry, the measuring was repeated until the maximum deviation was under 4 µm. The reported maximum deviation was 2µm.

### Liner classification

To develop a new liner coverage classification, liners with an uneven rim had to be covered in addition to neutral liners. 2 additional liners with a differing rim shape were included to be used as an example: a Continuum Longevity “elevated rim” liner and a Pinnacle Marathon “+4 10-degree face changing” liner. The Continuum elevated rim liner was measured with CMM, but we referred to the manufacturer’s specifications for the Pinnacle face changing liner, as its coverage is stated to be the same as with Marathon neutral liners. To find existing information and clinical results that could be used in developing a liner classification, a literature search was done. Ovid Medline, PubMed, and Google were used with keywords: hip, arthroplasty/replacement, liner, coverage, jumping distance, femoral head offset/inset, articular arc, and head center offset/inset.

Equator network guidelines were not directly applicable to this study as this is a descriptive study of single implants and human subjects were not involved.

### Outcomes

**Figure 1:**
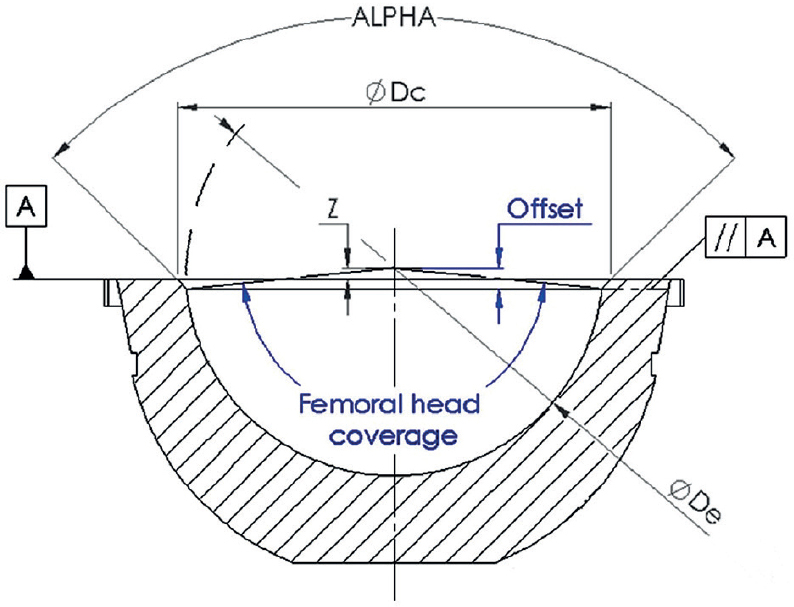
Liner section drawing. Coordinate measuring machine (CMM) measurements represented in grey (ALPHA: cone angle, Dc: circumference diameter, De: sphere diameter, Z: sphere offset over the planar surface A) and the measurements evaluated through the mathematical model represented in blue (femoral head offset, femoral head coverage).

CMM was used to measure the inner sphere diameter (De), the ubication of the center of rotation from the sphere (Z), the chamfer angle (ALPHA), and the diameter of the circumference (Dc) that mates the end of the chamfer with the exterior surface and is parallel to the top surface (A) (Figure 1).

To calculate the coverage of each liner (inner half-sphere in contact with the femoral head), the distance between the center of the femoral head and the opening plane, where the femoral head’s contact with the liner’s surface ends and the chamfer starts, was evaluated. This distance is called femoral head offset (FHO) or inset when the distance is negative [11,14]. As the direct measurement of the FHO and coverage with a CMM is not possible, a mathematical model was developed in order to gain the required measurements with the values obtained from the CMM measurements.

### Mathematical model

Neutral liners have axial symmetry; tested with the CMM, and due to the symmetry, the liner can be defined in a section.

The mathematical model comes from solving the equation of a line formed by the chamfer angle (APLHA/2 = α/2) and the circumference diameter (Dc), with the sphere formed by the hollow sphere diameter (De) and the distance of the hollow sphere center to the face plane of the liner (Z).


Femoral head coverage​(°)=2*acos(Offset/(De/2))*(180/π) 1

Offset (mm)=Z+(2*a*Dc–2*Z–sqrt((2*a*Dc–2*Z)^2–4*b*[Dc/(tan(α/2)*tan(90–α/2))^2–De^2+Z^2]))/(2*b) 2

a=1/(tan(α/2)*tan(90–α/2)^2) 3

b=(1+tan(90-α/2)^2)/(tan(90–α/2)^2) 4

### Data sharing, funding, and disclosures

The data presented in this study is available on request from the corresponding author. This study was supported by competitive research funding from Pirkanmaa Hospital District, representing governmental funding. The first author (JO) also received a personal research grant from the Finnish Arthroplasty Society for this research project. The authors declare no conflict of interest. Complete disclosure of interest forms according to ICMJE are available on the article page, doi: 10.2340/17453674.2024.41946

## Results

We found a marked 27.1° (range, 167.7° to 194.8°) variation in the coverage of the liners. The design with the lowest coverage was the Continuum Longevity (range 167.7°–169.3°), whereas the X3 liners had the highest coverage (range 192.2°–194.8°) (Table 1). 3 of the studied liners (Continuum, R3, and G7) had less than hemispherical coverage, whereas the coverage of the X3 was over a hemisphere by far (192.2°–194.8°).

**Table 1:** Coverage evaluation results for different neutral liner designs

Model and sizes	Coverage (°)
**Cup size 58 mm**	
Head size 36 mm	
X3 F	192.2
Pinnacle Marathon	185.5
G7 E1	176.9
R3	175.3
Continuum Longevity	168.9
Head size 32 mm	
X3 F	194.8
Pinnacle Marathon	180.0
G7 ArComXL	176.1
R3	175.4
Continuum Longevity	169.3
**Cup size 52 mm**	
Head size 36 mm	
X3 E	192.3
Pinnacle ALTRX	182.3
G7 E1	176.7
R3	176.0
Continuum Longevity	168.8
Head size 32 mm	
X3 E	194.8
Pinnacle Marathon	180.1
G7 E1	176.3
R3	175.1
Continuum Longevity	169.1
**Cup size 46 mm**	
Head size 32 mm	
X3 C	193.7
G7 E1	175.9
Head size 28 mm	
Pinnacle Marathon	179.9
R3	175.0
Continuum Longevity	167.7

X3: Stryker.

Pinnacle ALTRX: DePuy Synthes.

Pinnacle Marathon: DePuy Synthes.

G7 E1: Zimmer Biomet.

G7 ArComXL: Zimmer Biomet.

R3: Smith & Nephew.

Continuum Longevity: Zimmer Biomet.

### Coverage of each liner design

**Figure 2:**
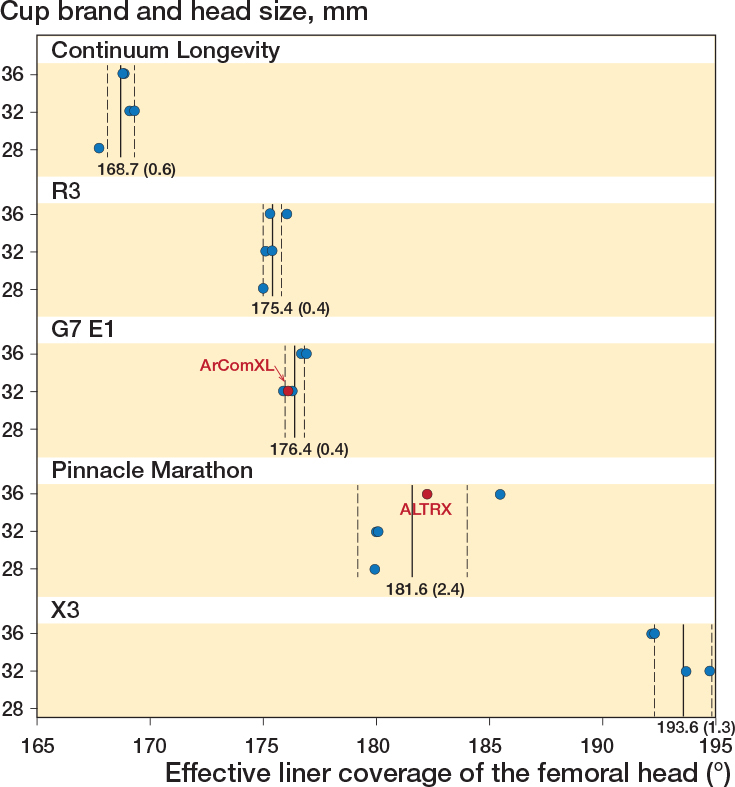
Evaluated effective coverage of the 25 different liners in the study clustered per manufacturer with vertical lines showing mean and dashed lines standard deviation.

Variations were also detected between the different sizes of each liner design (Figure 2). The biggest variations were detected with the Pinnacle (179.9°–185.5°) and X3 (192.2°–194.8°) liners, which were also the liners offering the largest coverage. The only overlapping in the coverage between the different liner designs was observed with the R3 (range 175.0°–176.1°) liner, which had the highest coverage (176.1° in size 52/32 mm), and the G7 (range 175.9°–176.9°) liner, which had the lowest coverage (175.9° in size 46/32 mm). In the Pinnacle liners with a 36 mm head size, the coverage increased with increasing cup size; the coverage of the ALTRX 52/36 mm was 182.3°, while that of the Marathon 58/36 mm was 185.5°. In the other liner designs and even in the Marathon liners for 32 mm heads, no increase in coverage with increasing cup size was observed or the increase was minute (0.3° in the R3 with 32 mm heads). The coverages of the ALTRX (182.3°) and the ArComXL (176.1°) liners were within the range of the rest of the Pinnacle and G7 liners.

### Continuum elevated rim liner

**Figure 3:**
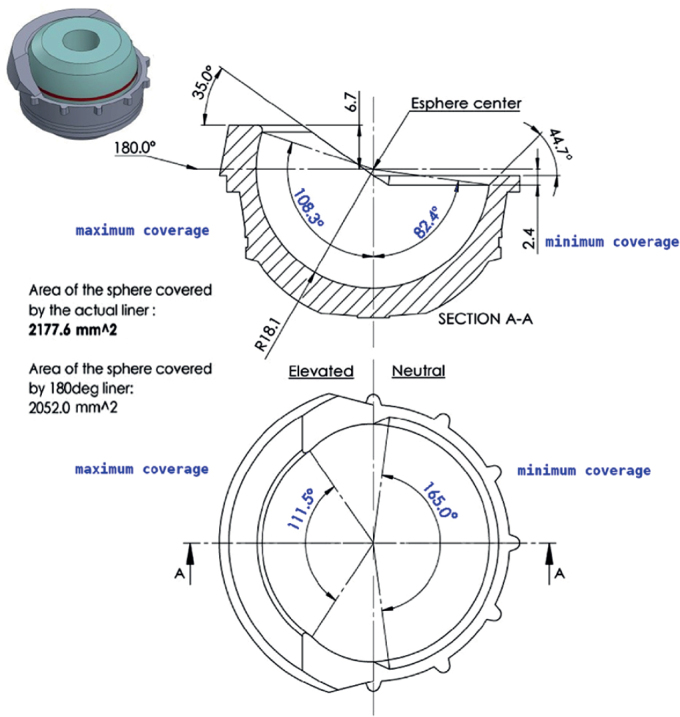
A sectional drawing of the Continuum elevated rim liner. The angles of the maximum and minimum coverage areas of the articulating surface are represented in blue.

The coverage of the Continuum Longevity elevated rim liner was 190.8° in the area with the rim elevation. The highest and lowest coverage portions of the liner constituted 111.5° and 165° of the rim circumference, respectively. The bevel between the elevated and neutral parts of the liner constituted 88.5° of the rim perimeter (Figure 3).

### Liner classification

The literature search revealed that clinically relevant results concerning liner coverage are currently insufficient to develop a clinically validated novel liner classification. As a first step to consider the liner coverage differences there should be a way to code the 3D geometry. There are basically 3 kinds of non-constrained liners available: neutral, elevated rim, and face changing liners. FHO would give sufficient information to describe the coverage of neutral liners, but more comprehensive information is required to cover the differences in the design of the articulating portion of the liner rim with elevated rim and face changing liners. To enable the comparison of coverages of different types of liners, we chose to present a set of descriptive parameters called “hemispheric coverage index values.” The idea is to compare the coverage of liners with a liner that would have a neutral rim and a completely hemispherical 180° coverage. The index values consist of 4 features (Table 2, Figure 3):

**Table 2:** Hemispheric coverage index values of the study liners. Hemispheric coverage (%) at 360° unless specified

Model	Total	Maximum	Minimum	Face change
X3 neutral	107.6	107.6	107.6	0°
Continuum elevated rim	106.1	120.0	91.6	0°
		at 111.5°	at 165.0°	
Pinnacle +4 10-degree	101.1	101.1	101.1	10°
Pinnacle neutral	101.1	101.1	101.1	0°
G7 neutral	98.0	98.0	98.0	0°
R3 neutral	97.4	97.4	97.4	0°
Continuum neutral	93.7	93.7	93.7	0°

1. Total hemispheric coverage (%), which is the liner contact area with the prosthesis head divided by the contact area of a hemispheric liner.
2. Maximum coverage (%, angle), which is the angle of the liner’s maximum coverage on the highest point of the cross-sectional half of the liner divided by 90° and the angle of maximum coverage on the rim.
3. Minimum coverage (%, angle), which is the angle of the liner’s minimum coverage on the lowest point of the cross-sectional half of the liner divided by 90° and the angle of minimum coverage on the rim.
4. Face change in degrees, which is when the liner rim is neutral, and the orientation of the opening plane is turned.

If the elevation of the rim were to change in a curvilinear manner, only the highest and lowest points of the rim, without an angle, would be reported and the total hemispheric coverage would still give more information regarding the coverage.

## Discussion

We aimed to analyze neutral liners from 4 major hip implant manufacturers. Marked differences were observed in the coverage of the liners, varying from 167.7° to 194.8°. In general, the variations between different cup and head sizes within each liner design were smaller than those between different brands. Our results reveal that each liner classified as neutral by the manufacturer offered a distinct design. Therefore, the term “neutral” does not tell the surgeon anything about the real coverage of the liner. As coverage directly affects both the range of motion (ROM) and, more importantly, the risk of THR dislocation, it is the true coverage that surgeons are really interested in. After all, the surgeon must balance the risks and benefits of these attributes when selecting the optimal THR components for an individual patient. However, because information on the liner coverage is not publicly available to all surgeons, patients undergoing THR are predisposed to unnecessary complications [6].

This is the first study to present an evaluation of the real coverage of commercially available PE liners. The only other study on assessment of liner coverage dealt with metal-on-metal (MoM) articulations [13].

While neutral liners are widely used [15], they are clearly not equal in terms of their actual design. The large differences in the liner coverage observed in our study predict completely different biomechanical behavior for the implants at both extremes. Although dislocation is a multifactorial phenomenon, the articulating surface of a liner is at the core of the stability of a THR, as liner coverage is the geometrical factor with the highest influence on JD [11]. Even though there is a scarcity of literature, coverage differences seem to be clinically meaningful as more dislocation complications have been reported with liners with smaller coverage in both clinical series and in a retrieval study [6,12,14]. In our study, the Continuum Longevity neutral liner had the smallest coverage. At a recent study from our institution, the 1-year dislocation rate for the Continuum Longevity neutral liner was 5.1%, whereas for the Pinnacle neutral liner it was 1.3% and for the Continuum Longevity elevated rim liner 1.2% [6]. According to our results, a 13° mean difference in the coverage between the Continuum and the Pinnacle neutral liners led to a 3.9 times higher dislocation rate in real-life clinical use. Dislocation rates, which discriminate between different liner types, have not been reported for the rest of the cup systems assessed in the current study. In the Australian Joint Replacement Registry, the Continuum has evinced higher dislocation revision rates than other THRs, and dislocation is also the most common reason for revision with the Continuum cup system [1].

The current use of the term “neutral” with liners that do not have rim elevation may give a false impression that all of these liners would have hemispherical coverage. The only manufacturer that gives information concerning the actual liner coverage in the operative technique manual is DePuy Synthes (the Pinnacle cup system): the manual describes that the Pinnacle neutral liners have 180° coverage. In our measurements, however, the coverage varied between 179.9° and 185.5°.

As an example of how to discriminate between liners on the basis of the varying coverage designs, “Hemispheric coverage index values” was presented. We believe surgeons need this kind of practical coverage information to better understand and to compare their implants when making clinical decisions. In future studies, one should search for clinically relevant coverage thresholds with regards to THR stability and impingement free ROM etc.

### Limitations

First, we did not measure liners in every possible cup and head size or with every available type of PE for the selected cup systems. Second, we measured only 1 liner of each selected size. Due to these limitations, our results are exposed to random variation and should be extrapolated with caution. We observed an increase in coverage with Pinnacle liners for 36 mm heads with increasing cup size. The fact that the measurements were double checked rules out measurement error. Based on our results, one cannot be sure whether the increase is a design feature, although the most probable explanation is that the Marathon liner in size 58/36 mm was faulty. This is supported by the notion that the coverage of Marathon liners for 32 mm heads remained constant and that the coverage of that liner (185.5°) appeared as an outlier value compared with the rest of the Pinnacle liners (179.9°–182.3°). The 5.6° variation in the coverage of Pinnacle liners was assumed to have been caused by the greater size of the chamfer and its high error tolerances, as it appeared to have been manufactured with a milling machine or lathe after the PE injection at the end of the fabrication process. Third, we did not measure the Pinnacle Marathon “+4 10-degree face changing” liner with CMM. As the main focus was on the coverage differences with neutral liners, the sole importance of the additional liners was to set an example of how to utilize the new liner coverage classification. Hence, the exact coverage information on these liners is of minor importance and purely an imaginary face changing liner might have been used for this purpose as well.

### Strengths

The independence of our study can be considered a strength, as this study was not financially supported by any of the manufacturers. The implants were purchased by our hospital for use in our daily practice. Furthermore, the accuracy of the CMM measurements is more than sufficient to detect differences in the articulating surfaces. Our results confirm reliable design differences in neutral PE liners, although only a selected cross-section of the products was studied.

### Conclusion

We found distinct differences in neutral classified PE liners of the most implanted acetabular cups used in contemporary THR. Based on our results it is clear that all neutral liners are not equal. We suggest our set of descriptive parameters called “hemispheric coverage index values” be used in discriminating the differences in liner coverage.

In perspective, we did not assess the rationale behind the design of each of the liners studied, as our primary aim was to investigate whether differences in the coverage of these liners exist and to raise awareness within the orthopedic community of the possible differences in design features. In future, a more transparent acknowledgment of liner coverage features might help to further enhance the results of THRs [3].

## Notes

All authors: methodology, interpretation of results, manuscript editing, and approval of the final version. JO: liner coverage evaluation and mathematical model, writing of the first version of the manuscript. PN: original planning of the study, hemispheric coverage index values, writing of the first version of the manuscript. JH, JV, AE: project supervision.

The authors would like to thank Jaakko Kotiranta from Metrology Laboratory Services, Tampere University, for the work with the measurements and Peter Heath for linguistic help.

Handling co-editors: Ivan Hvid and Robin Christensen

*Acta* thanks Claus Varnum for help with peer review of this manuscript.
